# Comment on Jankiewicz et al. The Effect of Goat-Milk-Based Infant Formulas on Growth and Safety Parameters: A Systematic Review and Meta-Analysis. *Nutrients* 2023, *15*, 2110 †

**DOI:** 10.3390/nu15214558

**Published:** 2023-10-27

**Authors:** Jan Mazela, Anna Bartnicka, Sophie Gallier

**Affiliations:** 1 Department of Neonatology, Poznan University of Medical Sciences, 33 Polna Street, 60-535 Poznan, Polandannabartnicka@ump.edu.pl (A.B.); 2 Dairy Goat Co-operative (N.Z.) Ltd., Hamilton 3240, New Zealand

We have read the article entitled “The effect of goat-milk-based infant formulas on growth and safety parameters: a systematic review and meta-analysis” by Jankiewicz et al. published in *Nutrients* [1].

We acknowledge the relevance of this systematic review due to the increasing number of goat milk formulas (GMFs) available worldwide. Only two GMFs have been clinically evaluated to help caregivers and healthcare professionals make an informed choice when selecting a nutritional product. However, the article did not present key compositional differences between the two GMFs, which may affect nutritional and health outcomes beyond infant growth. One formula evaluated by Grant et al. [2] and Zhou et al. [3] is based on whole goat milk as a source of proteins and fat (including milk fat globule membrane and *sn-2* palmitic acid), while the other formula tested by Xu et al. [4] and He et al. [5] is made from skim milk powder and whey protein powder as the sources of proteins and a vegetable oil blend as the sole source of fat.

In addition, a few issues appear unclear and should be corrected. The introduction refers to a higher level of α_s2_-casein as the protein linked to less protein aggregation in goat milk when it should refer to a lower level of α_s1_-casein. The risk of the bias assessment process for D1 (randomization process) is not clearly explained, and, based on information provided in all publications, it is unclear why the publication of Zhou et al. [3] was assessed as having “some concerns”. It is also not apparent how some risks for all domains were identified in some but not other publications. Table 1 (1) is missing some information from Zhou et al. [4] (stool and allergy measures were reported in Tannock et al. [6] and the cited publication, respectively), (2) includes an error as infants in this trial received formula until 12 months of age, not up to 4–12 months, and (3) should say “yes” in the column “published study protocol available” as the Grant et al. [3] publication includes all protocol information similar to the other three publications.

Furthermore, Section 4.2 should be corrected as the mention of goat milk as a source of proteins and fatty acids is only valid for studies on the formula based on whole goat milk. The reference to additional studies should indicate which formula (whole milk-based or whey-adjusted, vegetable oil-only GMF) was used. And the reference to two ongoing trials should also indicate which formula is used, as the nutritional and health outcomes in these two studies are only valid for the formula under investigation. For example, it is well known that for allergy outcomes, clinical data and efficacy in allergy prevention or treatment of hydrolysed formulas cannot be generalised to any hydrolysed formulas. Therefore, the same would apply to the allergy outcome results from the GIraFFE study (NCT04599946).

Importantly, a statement about the conflicts of interest from the GMF company authors is missing.
